# Development and validation of a predictive model for rapid renal function decline in early IgA nephropathy: a multicenter retrospective study

**DOI:** 10.3389/fmed.2026.1857665

**Published:** 2026-09-24

**Authors:** Junan Zhou, Yang Liu, Lixia Huo, Xu Wu, Zhelun Zhou, Haodong Liu, Rong Wu, Jiaai Zhang, Yiqing Yu, Deyong Fan, Xiaoyi Wang

**Affiliations:** 1 HuZhou Postgraduate Joint Training Base, Zhejiang Chinese Medical University, China; 2 Huzhou Key Laboratory of Chronic Kidney Disease, First Affiliated Hospital, Huzhou University, Huzhou, China; 3 Department of Nephrology, The First Affiliated Hospital of Huzhou University, Huzhou, China; 4 Department of Nephrology, Huzhou Central Hospital, Huzhou, China

**Keywords:** IgA nephropathy, lambda light chain, Oxford C score, prediction model, rapid renal function decline

## Abstract

Immunoglobulin A nephropathy (IgAN) is a heterogeneous disease, and early identification of patients at high risk of rapid renal deterioration remains challenging. In this multicenter retrospective study, 483 patients with biopsy-proven IgAN at chronic kidney disease stages 1–3 were enrolled and randomly assigned to a training cohort (*n* = 338) and an internal validation cohort (*n* = 145). Rapid renal function decline was defined as a decline of at least 25% in estimated glomerular filtration rate or progression to ESRD, analyzed as a time-to-event outcome from renal biopsy (the 1-year risk was the principal metric reported). Independent predictors were identified using Cox proportional hazards regression, and a nomogram was developed and internally validated. In the training cohort, 83 patients (24.6%) reached the endpoint during follow-up (median time to event 13.6 months). Multivariable analysis showed that renal tissue Lambda light chain deposition, higher systolic blood pressure, serum chloride, blood urea nitrogen, and Oxford C score were independent risk factors, whereas higher serum complement C3 and potassium were protective factors. The nomogram showed good discrimination, with a 1-year time-dependent area under the receiver operating characteristic curve (AUC) of 0.86 (95% CI: 0.81–0.92) in the training cohort and 0.80 (95% CI: 0.69–0.91) in the validation cohort. SHAP analysis identified Lambda light chain deposition as the most influential predictor. In conclusion, we developed and internally validated a nomogram integrating clinicopathological variables to predict rapid renal function decline in early-stage IgAN, which may facilitate early risk stratification and individualized management.

## 1 Introduction

Immunoglobulin A nephropathy (IgAN) is the most prevalent primary glomerular disease worldwide and represents one of the leading causes of chronic kidney disease (CKD) and end-stage renal disease (ESRD) (1). The prevalence of IgAN varies significantly by region, with a notably higher disease burden observed in Asian populations (2). This disease exhibits marked differences in disease progression and pathological manifestations, resulting in considerable variability in patient prognosis. Epidemiological data indicate that approximately 30% to 40% of confirmed cases may progress to end-stage renal disease within 10 to 20 years (3).

According to the 2025 International Clinical Practice Guidelines for Kidney Disease: Improving Global Outcomes (KDIGO), sustained reduction of proteinuria to <0.5 g/day following supportive therapy is a key prognostic indicator and an evidence-based criterion for guiding therapeutic decisions in patients with IgAN (4). However, in clinical practice, some patients exhibit unfavorable clinical and pathological features before reaching this target, and a considerable proportion still fail to achieve it despite standardized treatment (5). Therefore, accurately identifying high-risk individuals during early disease stages (CKD 1–3) is imperative for timely intervention.

Although numerous prognostic models for IgAN exist, most focus on long-term outcomes such as progression to end-stage renal disease (ESRD) (6). Tools specifically designed to predict rapid, short-term progression are lacking. Recent mechanistic work has suggested that renal immunoglobulin light chain deposition, particularly of the lambda isotype, contributes to IgAN pathogenesis via enhanced mesangial trapping and lectin-pathway complement activation (3). However, whether mesangial lambda deposition independently predicts short-term rapid decline — beyond established clinical and Oxford MEST-C variables — has not been systematically examined (7).

This study aims to evaluate clinical and pathological data through a retrospective analysis to identify risk factors associated with the rapid progression of IgAN. Furthermore, we seek to develop a nomogram model to assist clinicians in early identification of high-risk patients, thereby providing a reliable foundation for timely and individualized intensified treatment strategies.

## 2 Methods

### 2.1 Research design and participants

The main study cohort consisted of 483 adult patients (aged ≥18 years) diagnosed with primary IgAN via renal biopsy at The First Affiliated Hospital of Huzhou University and Huzhou Central Hospital between January 2013 and December 2023. Inclusion criteria were as follows: (1) a confirmed diagnosis of primary IgAN by renal biopsy; (2) presence of chronic kidney disease (CKD) stages 1–3 at the time of diagnosis, as defined by standard CKD diagnostic criteria (41); (3) availability of complete baseline clinical, laboratory, and histopathological data. Exclusion criteria included: (1) secondary IgAN (e.g., associated with lupus nephritis, Henoch-Schonlein purpura nephritis, or hepatitis); (2) estimated glomerular filtration rate (eGFR) <30 mL/min/1.73 m^2^; (3) fewer than eight glomeruli observed under light microscopy in the biopsy specimen or absence of a biopsy report; (4) incomplete baseline clinical, laboratory, or histopathological data precluding model covariates or outcome ascertainment. To minimize selection bias, all patients meeting the prespecified eligibility criteria at each participating center were enrolled consecutively throughout the study window (2013–2023); no patients were excluded beyond the prespecified analytic criteria listed above. The study was conducted in accordance with the Declaration of Helsinki. A flowchart of patient screening, eligibility assessment, and cohort allocation is shown in Figure 1.

**Figure 1:**
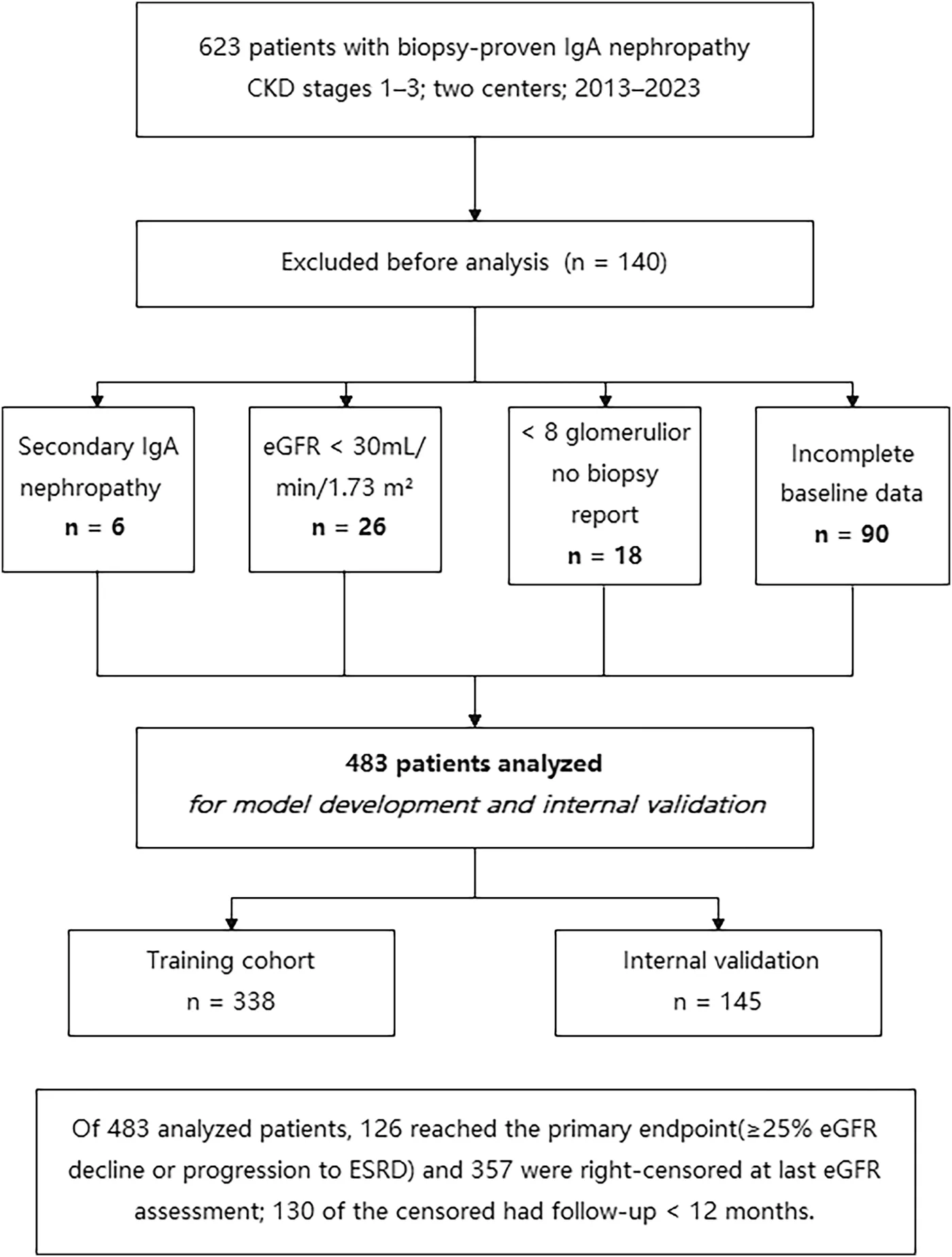
Flowchart onf patient screening, eligibility assessment, and cohort allocation. Of the 483 patients analyzed, 126 reached the primary endpoint (≥25% eGFR decline or progression to ESRD) and 357 were right-censored at the last eGFR assessment, including 130 with follow-up <12 months.

### 2.2 Data collection and variables

The baseline data for all patients were retrospectively collected from the electronic medical record system and included the following categories: demographic characteristics (age and gender); clinical parameters (systolic blood pressure [SBP], diastolic blood pressure [DBP], history of hypertension, and smoking history); and laboratory measurements. Laboratory samples were obtained within one week prior to renal biopsy and included 24 h urine protein quantification, serum albumin, serum creatinine (Scr), blood urea nitrogen (BUN), uric acid (UA), serum potassium (K), sodium (Na), chloride (Cl), calcium (Ca), magnesium (Mg), hemoglobin, C-reactive protein (CRP), and complement C3 and C4 levels. Pathological evaluations were performed on all renal biopsy specimens, which were independently assessed by two senior renal pathologists blinded to the clinical information. The pathological assessment followed the Oxford classification system and included evaluation of mesangial hypercellularity (M0/M1), endocapillary hypercellularity (E0/E1), segmental glomerulosclerosis (S0/S1), tubular atrophy and interstitial fibrosis (T0–T2), and crescent formation (C0/C1/C2). Additionally, deposition of kappa and lambda light chains in renal tissues was specifically evaluated. The majority of patients received standard-of-care treatment, including renin–angiotensin system (RAS) inhibitors and/or corticosteroids. Because treatment was assigned according to guideline-directed care rather than randomized, and was therefore confounded by disease severity, treatment variables were not included as predictors in the final model.

Direct immunofluorescence was used to evaluate deposition of kappa and lambda light chains in renal tissues. Four-micrometer formalin-fixed, paraffin-embedded sections were incubated with FITC-conjugated monospecific antisera against human kappa and lambda light chains and anti-IgA; mesangial staining was graded 0–3+ independently by two senior renal pathologists blinded to clinical data. For the prediction model, renal tissue lambda light chain deposition was coded as a binary variable, with presence defined as ≥1+ mesangial staining and absence as no detectable staining. Notably, the kappa-to-lambda isotype ratio was not specifically quantified; therefore, “lambda deposition” in this study denotes the presence of mesangial lambda staining (≥1+), independent of the kappa/lambda dominance ratio or light-chain restriction pattern.

### 2.3 Outcome definition

The primary outcome was defined as rapid renal function decline, characterized by a decline in baseline eGFR of 25% or greater or progression to end-stage renal disease (ESRD), analyzed as a time-to-event outcome from renal biopsy (the 1-year risk was the principal metric reported). Patients were classified into “high-risk group” and “low-risk group” based on whether this endpoint was met (the training-cohort stratification is presented in Table 2).

Rationale for the 25% eGFR-decline endpoint: although *a* ≥ 50% eGFR decline or ESRD is the conventional IgAN endpoint, our median follow-up of approximately 14 months yielded few hard ESRD events; to improve statistical power without losing prognostic relevance, we adopted *a* ≥ 25% eGFR decline as a more conservative variant of the ≥30% early-progression endpoint that has been increasingly accepted and validated in IgAN (8).

### 2.4 Statistical analysis and model construction

Statistical analyses were conducted using R software (version 4.4.1) and Zstats v1.0. Data partitioning: The final cohort of 483 patients was randomly allocated to a training cohort (*n* = 338) and an internal validation cohort (*n* = 145) at a 7:3 ratio, which facilitated model development and internal validation. Baseline characteristic comparisons: In the training cohort, categorical variables were summarized as frequencies and percentages, with group comparisons performed using the chi-square test or Fisher's exact test, as appropriate. Continuous variables with normal distribution were reported as mean ± standard deviation and compared using the independent samples *t*-test. Non-normally distributed continuous variables were presented as median (interquartile range) and analyzed using the Mann–Whitney *U*-test. Predictor selection and model development: Within the training cohort, univariate Cox proportional hazards regression analysis was initially applied to identify variables significantly associated with rapid progression (*P* < 0.05). Subsequently, statistically significant variables were incorporated into a multivariable Cox proportional hazards regression model with stepwise selection to determine independent predictors. Variables included in the multivariable model were selected based on both univariable statistical significance and clinical as well as pathological relevance. To reduce model complexity and the risk of overfitting, only a limited number of predictors were retained in the final model. Based on the final multivariate model, a nomogram for predicting the 1-year risk of rapid progression was developed using the rms package in R.To limit overfitting, the final multivariable model retained a parsimonious set of predictors, yielding an events-per-variable ratio (training events/predictors∼12) well above the conventional threshold of 10. No missing values were present in any predictor or outcome variable; complete-case analysis was therefore applied without imputation. For the time-to-event analysis, follow-up time was defined as the interval from renal biopsy to the primary endpoint (≥25% eGFR decline or progression to ESRD); patients without an observed endpoint were right-censored at their last eGFR assessment. In the training cohort, 83 patients (24.6%) reached the endpoint and 255 were censored (median follow-up 15.2 months, range 6.0–83.9); in the validation cohort, 43 (29.7%) reached the endpoint and 102 were censored (median follow-up 13.4 months, range 6.0–94.1).

Sensitivity and subgroup analyses: To evaluate whether the association between renal tissue Lambda light chain deposition and rapid progression was confounded by baseline renal function or therapeutic regimen, we re-fitted the final multivariable Cox model in the training cohort with additional adjustment for (i) eGFR, (ii) CKD stage (stage 2/3 vs. stage 1), and (iii) use of RAS inhibitors, glucocorticoids, and immunosuppressants. We further tested for effect modification by including product interaction terms between Lambda deposition and CKD stage or medication use, using likelihood-ratio tests. Exploratory subgroup analyses of the Lambda–outcome association were additionally performed on the full cohort (*n* = 483) across strata defined by CKD stage, medication use, and eGFR.

### 2.5 Model validation and evaluation

Discrimination was evaluated by computing the 1-year time-dependent AUC (at the 12-month time point of the time-to-event model) for both the training and validation cohorts. Calibration was assessed by generating calibration curves comparing predicted and observed probabilities. Clinical utility was examined through decision curve analysis (DCA), which estimated the net benefit of the model across a range of threshold probabilities. All statistical analyses were conducted using two-sided tests, with statistical significance defined as *p* < 0.05. To improve interpretability of the final model, SHAP (SHapley Additive exPlanations) analysis was performed in the training cohort. SHAP values were calculated for all predictors in the final model, and variable importance plots as well as summary plots were generated to visualize the relative contribution and distribution of each feature in the final nomogram model.

Internal validation was further supported by bootstrap resampling (1,000 repetitions) to derive optimism-corrected discrimination and calibration. Because the training and validation sets were obtained by random split of a single pooled cohort, the above validation is internal; external multicenter validation is planned.

## 3 Results

### 3.1 Study population and baseline characteristics

A total of 483 patients with biopsy-proven IgA nephropathy (CKD stages 1–3) were enrolled and randomly stratified into a training cohort (*n* = 338) and an internal validation cohort (*n* = 145) at a ratio of 7:3. The baseline characteristics were well-balanced between the training and validation cohorts, with no statistically significant differences observed (Table 1). In the training cohort, 83 patients (24.6%) met the primary endpoint of rapid renal function decline (≥25% eGFR decline or ESRD) during follow-up and were classified as the high-risk group. The remaining 255 patients (75.4%) comprised the low-risk group. Comparative analysis demonstrated distinct clinical profiles between the two groups (Table 2). The high-risk group exhibited significantly elevated systolic blood pressure (SBP), diastolic blood pressure (DBP), age, serum uric acid, 24 h urinary protein, serum creatinine, blood urea nitrogen (BUN), and serum chloride levels compared to the low-risk group. Conversely, baseline eGFR, serum albumin, and serum calcium levels were significantly lower in the high-risk group. Pathologically, patients with rapid progression showed a higher prevalence of Lambda and Kappa light chain deposition, as well as higher rates of Oxford classification lesions, specifically mesangial hypercellularity (M1), endocapillary hypercellularity (E1), and tubular atrophy/interstitial fibrosis (T1/2).

**Table 1:** Balance assessment between the training and validation cohorts.

Variables	Total (*n* = 483)	Validation (*n* = 145)	Training (*n* = 338)	*P* value
Age (years)	42.00 (32.00, 52.00)	41.00 (32.00, 52.00)	42.00 (32.00, 52.00)	0.781
Weight (kg)	63.10 (56.00, 71.70)	64.50 (59.00, 73.00)	63.00 (55.60, 70.97)	0.078
SBP (mmHg)	133.00 (122.00, 149.00)	135.00 (123.00, 150.00)	132.00 (121.00, 148.00)	0.255
DBP (mmHg)	84.22 ± 13.14	84.82 ± 12.49	83.97 ± 13.41	0.514
BMI (kg/m^2^)	23.98 (21.24, 26.24)	24.22 (21.97, 26.40)	23.56 (21.08, 26.12)	0.144
Hemoglobin (g/L)	128.64 ± 19.86	128.47 ± 20.76	128.71 ± 19.49	0.904
Serum uric acid (*μ*mol/L)	381.62 ± 100.78	382.19 ± 112.76	381.38 ± 95.35	0.935
Follow (months)	13.75 (10.51, 23.42)	13.00 (10.23, 22.17)	14.25 (11.08, 24.35)	0.126
Proteinuria (mg/day)	1467.30 (871.80, 2555.25)	1397.00 (870.00, 2131.80)	1474.75 (873.70, 2831.88)	0.281
eGFR(mL/min/1.73m^2^)	76.59 (57.21, 98.22)	77.68 (61.95, 93.24)	75.77 (55.68, 99.64)	0.712
Albumin (g/L)	36.30 (33.10, 40.30)	37.10 (33.20, 41.00)	35.90 (33.10, 39.88)	0.179
Serum creatinine (μmol/L)	86.00 (68.45, 112.10)	86.20 (68.60, 108.80)	84.75 (68.10, 112.55)	0.919
CRP (mg/L)	0.70 (0.50, 2.10)	0.70 (0.50, 2.20)	0.60 (0.50, 2.00)	0.983
Serum C3 (g/L)	0.93 (0.79, 1.12)	0.94 (0.82, 1.09)	0.93 (0.77, 1.13)	0.884
Serum C4 (g/L)	0.23 (0.19, 0.30)	0.23 (0.19, 0.30)	0.23 (0.19, 0.30)	0.939
K (mmol/L)	3.88 (3.63, 4.14)	3.92 (3.66, 4.17)	3.85 (3.62, 4.14)	0.398
Na (mmol/L)	140.80 (139.35, 142.40)	141.10 (139.70, 142.30)	140.60 (139.22, 142.40)	0.176
Cl (mmol/L)	107.20 (105.40, 108.95)	107.20 (105.70, 109.00)	107.15 (105.30, 108.90)	0.687
Ca (mmol/L)	2.21 (2.12, 2.32)	2.21 (2.12, 2.33)	2.21 (2.12, 2.32)	0.738
Mg (mmol/L)	0.85 (0.79, 0.91)	0.84 (0.77, 0.93)	0.85 (0.79, 0.91)	0.573
Bun (mmol/L)	5.63 (4.71, 7.05)	5.69 (4.70, 7.05)	5.62 (4.72, 7.05)	0.97
Censored, *n* (%)	357 (73.9)	102 (70.3)	255 (75.4)	0.291
Follow-up of censored patients (months)	14.26 (10.56–24.47)	13.43 (10.28–22.39)	15.17 (10.78–25.12)	0.330
Sex, *n* (%)				0.156
Man	203 (42.03)	68 (46.90)	135 (39.94)	
Female	280 (57.97)	77 (53.10)	203 (60.06)	
Hypertension, *n* (%)				0.396
No	297 (61.49)	85 (58.62)	212 (62.72)	
Yes	186 (38.51)	60 (41.38)	126 (37.28)	
Diabetes, *n* (%)				0.567
No	471 (97.52)	140 (96.55)	331 (97.93)	
Yes	12 (2.48)	5 (3.45)	7 (2.07)	
Cigarette, *n* (%)				0.863
No	392 (81.16)	117 (80.69)	275 (81.36)	
Yes	91 (18.84)	28 (19.31)	63 (18.64)	
CKD, *n* (%)				0.056
Stage 1	165 (34.16)	46 (31.72)	119 (35.21)	
Stage 2	189 (39.13)	68 (46.90)	121 (35.80)	
Stage 3	129 (26.71)	31 (21.38)	98 (28.99)	
Kappa, *n* (%)				0.422
No	154 (31.88)	50 (34.48)	104 (30.77)	
Yes	329 (68.12)	95 (65.52)	234 (69.23)	
Lambda, *n* (%)				0.536
No	147 (30.43)	47 (32.41)	100 (29.59)	
Yes	336 (69.57)	98 (67.59)	238 (70.41)	
M, *n* (%)				0.77
0	32 (6.63)	8 (5.52)	24 (7.10)	
1	451 (93.37)	137 (94.48)	314 (92.90)	0.521
E, *n* (%)				0.465
0	418 (86.54)	128 (88.28)	290 (85.80)	
1	65 (13.46)	17 (11.72)	48 (14.20)	
S, *n* (%)				0.361
0	208 (43.06)	67 (46.21)	141 (41.72)	
1	275 (56.94)	78 (53.79)	197 (58.28)	
T, *n* (%)				0.744
0	390 (80.75)	120 (82.76)	270 (79.88)	
1	83 (17.18)	22 (15.17)	61 (18.05)	
2	10 (2.07)	3 (2.07)	7 (2.07)	
C, *n* (%)				0.375
0	309 (63.98)	94 (64.83)	215 (63.61)	
1	161 (33.33)	45 (31.03)	116 (34.32)	
2	13 (2.69)	6 (4.14)	7 (2.07)	

BMI, body mass index; SBP, systolic blood pressure; DBP, diastolic blood pressure; Scr, serum creatinine; BUN, blood urea nitrogen; UA, uric acid; CRP, C-reactive protein; eGFR, estimated glomerular filtration rate; CKD, chronic kidney disease; K, potassium; Na, sodium; Cl, chloride; Ca, calcium; Mg, magnesium; M0/M1, mesangial hypercellularity score; E0/E1, endocapillary hypercellularity score; S0/S1, segmental glomerulosclerosis score; T0/T1/T2, tubular atrophy/interstitial fibrosis score; C0/C1/C2, crescent score.

**Table 2:** Analysis of fundamental characteristics and differences.

Variables	Total (*n* = 338)	Low risk (*n* = 255)	High risk (*n* = 83)	*P* value
Age (years)	42.00 (32.00, 52.00)	41.00 (31.00, 51.00)	46.00 (34.50, 58.50)	0.001
Weight (rkg)	63.00 (55.60, 70.97)	62.00 (55.00, 70.00)	65.00 (58.55, 72.35)	0.037
SBP (rmmHg)	132.00 (121.00, 148.00)	129.00 (119.00, 142.00)	146.00 (127.50, 156.50)	<.001
DBP (rmmHg)	83.97 ± 13.41	82.53 ± 12.48	88.39 ± 15.20	<.001
BMI (rkg/m^2^)	23.56 (21.08, 26.12)	23.36 (20.96, 26.00)	24.42 (21.58, 26.56)	0.099
Hemoglobin (rg/L)	128.71 ± 19.49	128.87 ± 18.57	128.20 ± 22.20	0.787
Serum uric acid (μmol/L)	381.38 ± 95.35	370.41 ± 96.32	415.08 ± 84.30	<.001
Follow (rmonths)	14.25 (11.08, 24.35)	15.17 (10.78, 25.12)	13.57 (11.77, 20.62)	0.557
Proteinuria (mg/day)	1474.75 (873.70, 2831.88)	1331.50 (828.25, 2475.00)	2066.20 (1214.65, 4008.75)	<.001
eGFR(mL/min/1.73m^2^)	75.77 (55.68, 99.64)	78.17 (60.03, 102.48)	65.66 (44.72, 95.17)	0.001
Albumin (rg/L)	35.90 (33.10, 39.88)	36.30 (33.45, 40.90)	35.30 (31.60, 38.00)	0.012
Serum creatinine (μmol/L)	84.75 (68.10, 112.55)	80.00 (66.60, 109.70)	97.20 (74.65, 133.50)	<.001
CRP (rmg/L)	0.60 (0.50, 2.00)	0.80 (0.50, 2.20)	0.50 (0.50, 1.35)	0.211
Serum C3 (rg/L)	0.93 (0.77, 1.13)	0.93 (0.78, 1.15)	0.92 (0.76, 1.08)	0.293
Serum C4 (rg/L)	0.23 (0.19, 0.30)	0.23 (0.18, 0.29)	0.24 (0.21, 0.31)	0.168
K (rmmol/L)	3.85 (3.62, 4.14)	3.86 (3.64, 4.14)	3.85 (3.56, 4.10)	0.408
Na (rmmol/L)	140.60 (139.22, 142.40)	140.50 (139.20, 142.30)	140.80 (139.35, 142.75)	0.223
Cl (rmmol/L)	107.15 (105.30, 108.90)	107.00 (105.05, 108.60)	107.90 (106.00, 109.65)	0.002
Ca (rmmol/L)	2.21 (2.12, 2.32)	2.23 (2.13, 2.33)	2.19 (2.10, 2.25)	0.047
Mg (rmmol/L)	0.85 (0.79, 0.91)	0.85 (0.80, 0.91)	0.84 (0.79, 0.91)	0.619
Bun (rmmol/L)	5.62 (4.72, 7.05)	5.41 (4.56, 6.74)	6.44 (5.52, 8.28)	<.001
Sex, *n* (%)				0.005
Man	135 (39.94)	91 (35.69)	44 (53.01)	
Female	203 (60.06)	164 (64.31)	39 (46.99)	
Hypertension, *n* (%)				0.004
No	212 (62.72)	171 (67.06)	41 (49.40)	
Yes	126 (37.28)	84 (32.94)	42 (50.60)	
Diabetes, *n* (%)				0.114
No	331 (97.93)	252 (98.82)	79 (95.18)	
Yes	7 (2.07)	3 (1.18)	4 (4.82)	
Cigarette, *n* (%)				0.002
No	275 (81.36)	217 (85.10)	58 (69.88)	
Yes	63 (18.64)	38 (14.90)	25 (30.12)	
CKD, *n* (%)				0.009
Stage 1	119 (35.21)	96 (37.65)	23 (27.71)	
Stage 2	121 (35.80)	96 (37.65)	25 (30.12)	
Stage 3	98 (28.99)	63 (24.71)	35 (42.17)	
Kappa, *n* (%)				<.001
No	104 (30.77)	96 (37.65)	8 (9.64)	
Yes	234 (69.23)	159 (62.35)	75 (90.36)	
Lambda, *n* (%)				<.001
No	100 (29.59)	94 (36.86)	6 (7.23)	
Yes	238 (70.41)	161 (63.14)	77 (92.77)	
M, *n* (%)				0.044
0	24 (7.10)	22 (8.63)	2 (2.41)	
1	314 (92.90)	233 (91.37)	81 (97.59)	0.055
E, *n* (%)				0.009
0	290 (85.80)	226 (88.63)	64 (77.11)	
1	48 (14.20)	29 (11.37)	19 (22.89)	
S, *n* (%)				0.353
0	141 (41.72)	110 (43.14)	31 (37.35)	
1	197 (58.28)	145 (56.86)	52 (62.65)	
T, *n* (%)				<.001
0	270 (79.88)	220 (86.27)	50 (60.24)	
1	61 (18.05)	33 (12.94)	28 (33.73)	
2	7 (2.07)	2 (0.78)	5 (6.02)	
C, *n* (%)				0.092
0	215 (63.61)	170 (66.67)	45 (54.22)	
1	116 (34.32)	81 (31.76)	35 (42.17)	
2	7 (2.07)	4 (1.57)	3 (3.61)	

In this table, “high-risk” and “low-risk” groups refer to the training cohort (*n* = 338); high-risk indicates patients who met the primary endpoint during follow-up (*n* = 83), and low-risk indicates those who did not (*n* = 255).

### 3.2 Independent predictors of rapid progression

To identify potential predictors of rapid renal function decline, a univariate Cox proportional hazards regression analysis was first performed in the training cohort. This initial screening identified several variables significantly associated with the outcome (*P* < 0.05), including renal tissue Lambda light chain deposition, Oxford C classification (crescents), systolic blood pressure, serum complement C3, serum potassium, serum chloride, and blood urea nitrogen. These statistically significant variables were subsequently entered into a multivariable Cox regression model with stepwise selection to determine independent predictors. The final multivariable analysis yielded seven independent prognostic factors, comprising five risk factors and two protective factors (Table 3). Among the risk factors, renal tissue Lambda light chain deposition emerged as the strongest predictor of rapid progression, with a substantially elevated hazard ratio (HR) of 12.21 (95% CI: 4.56–32.69; *P* < 0.001). Other independent risk factors included Oxford C classification (C1 vs. C0: HR = 1.60, *P* = 0.045; C2 vs. C0: HR = 5.97, *P* = 0.005), elevated systolic blood pressure (HR = 1.02, *P* < 0.001), higher serum chloride (HR = 1.14, *P* = 0.002), and elevated blood urea nitrogen (HR = 1.10, *P* = 0.025). In contrast, higher serum complement C3 (HR = 0.23, *P* = 0.001) and higher serum potassium (HR = 0.33, *P* = 0.001) were identified as independent protective factors.

**Table 3:** Results of the univariate and multivariate Cox proportional hazards models.

Variables	Univariable analysis		Multivariable analysis	
	HR (95%CI)	*P* value	HR (95%CI)	*P* value
Age (ryears)	1.03 (1.02–1.05)	<.001		
Weight (rkg)	1.02 (1.01–1.04)	0.029		
SBP (rmmHg)	1.02 (1.01–1.03)	<.001	1.02 (1.01–1.03)	<.001
DBP (rmmHg)	1.03 (1.01–1.05)	<.001		
BMI (rkg/m^2^)	1.04 (0.98–1.10)	0.169		
Hemoglobin (rg/L)	0.99 (0.98–1.00)	0.091		
Serum uric acid (μmol/L)	1.01 (1.01–1.01)	<.001		
Proteinuria (mg/day)	1.01 (1.01–1.01)	<.001		
eGFR(mL/min/1.73m^2^)	0.99 (0.98–0.99)	0.01		
Albumin (rg/L)	0.95 (0.93–0.98)	<.001		
Serum creatinine (μmol/L)	1.01 (1.01–1.02)	<.001		
CRP (rmg/L)	1.00 (0.96–1.03)	0.895		
Serum C3 (rg/L)	0.21 (0.09–0.49)	<.001	0.23 (0.10–0.56)	0.001
Serum C4 (rg/L)	0.26 (0.02–3.28)	0.3		
K (rmmol/L)	0.50 (0.27–0.94)	0.03	0.33 (0.17–0.66)	0.001
Na (rmmol/L)	1.07 (0.98–1.17)	0.144		
Cl (rmmol/L)	1.25 (1.16–1.36)	<.001	1.14 (1.05–1.25)	0.002
Ca (rmmol/L)	0.09 (0.02–0.32)	<.001		
Mg (rmmol/L)	0.08 (0.01–0.73)	0.025		
Bun (rmmol/L)	1.18 (1.10–1.26)	<.001	1.10 (1.01–1.20)	0.025
Sex				
Man	1.00 (Reference)			
Female	0.57 (0.37–0.87)	0.01		
Hypertension				
No	1.00 (Reference)			
Yes	2.09 (1.36–3.23)	<.001		
Diabetes				
No	1.00 (Reference)			
Yes	1.98 (0.72–5.42)	0.183		
Cigarette				
No	1.00 (Reference)			
Yes	1.93 (1.20–3.08)	0.006		
CKD				
Stage 1	1.00 (Reference)			
Stage 2	0.88 (0.50–1.56)	0.668		
Stage 3	1.96 (1.15–3.33)	0.013		
Kappa				
No	1.00 (Reference)			
Yes	9.84 (4.40–22.01)	<.001		
Lambda				
No	1.00 (Reference)		1.00 (Reference)	
Yes	13.64 (5.34–34.85)	<.001	12.21 (4.56–32.69)	<.001
M, *n* (%)				
0	1.00 (Reference)			
1	4.68 (1.15–19.06)	0.031		
E, *n* (%)				
0	1.00 (Reference)			
1	2.63 (1.55–4.46)	<.001		
S, *n* (%)				
0	1.00 (Reference)			
1	1.16 (0.74–1.81)	0.515		
T, *n* (%)				
0	1.00 (Reference)			
1	2.54 (1.59–4.07)	<.001		
2	6.50 (2.58–16.38)	<.001		
C, *n* (%)				
0	1.00 (Reference)		1.00 (Reference)	
1	1.68 (1.08–2.63)	0.022	1.60 (1.01–2.54)	0.045
2	8.93 (2.67–29.86)	<.001	5.97 (1.73–20.53)	0.005

The distribution of major therapeutic regimens by progression status is shown in Table 4; renin-angiotensin system inhibitors, glucocorticoids, and immunosuppressants were all used more frequently in the progressor group, but because treatment was assigned per guideline-directed care rather than randomized, it was not included as a predictor.

**Table 4:** Distribution of major therapeutic regimens by progression status.

Therapeutic regimen	Total (*n* = 483)	Progressors (*n* = 126)	Non-progressors (*n* = 357)	*P* value
RAS inhibitors (ACEI/ARB)	311 (64.4%)	92 (73.0%)	219 (61.3%)	0.019
Glucocorticoids (prednisone/methylprednisolone)	175 (36.2%)	61 (48.4%)	114 (31.9%)	<0.001
Immunosuppressants (MMF/cyclophosphamide/rituximab)	148 (30.6%)	48 (38.1%)	100 (28.0%)	0.035
None of the above	62 (12.8%)	3 (2.4%)	59 (16.5%)	<0.001

Although 24 h proteinuria and baseline eGFR were strongly associated with the outcome in univariable analysis, they were not retained as independent predictors after adjustment; SHAP interpretability was therefore computed on the seven variables included in the final model.

### 3.3 Model development and validation

Based on the seven identified independent predictors, a prognostic nomogram was constructed to quantify the individual risk of rapid renal function decline at one year (Figure 2). The discriminative performance of the model was evaluated using the 1-year time-dependent AUC (AUC). The nomogram achieved an AUC of 0.86 (95% CI: 0.81–0.92) in the training cohort and 0.80 (95% CI: 0.69–0.91) in the internal validation cohort, indicating robust predictive accuracy (Figure 3). Bootstrap optimism correction (1,000 resamples) yielded an optimism-corrected AUC of 0.85 and a corrected calibration slope of 1.03 (apparent 1.00), indicating minimal overfitting and good calibration. Model calibration was assessed by comparing the predicted probabilities against the actual observed event frequencies. As illustrated in Figure 4, Calibration curves for both the training and validation cohorts demonstrated high consistency between the predicted probabilities and observed frequencies. Furthermore, Decision Curve Analysis (DCA) was employed to evaluate the clinical utility of the nomogram. The results (Figure 5) demonstrated that using the nomogram to guide clinical decisions yielded a higher net benefit compared to the default strategies of “treating all patients” or “treating no patients” across a broad range of threshold probabilities in both the training and validation cohorts.

**Figure 2:**
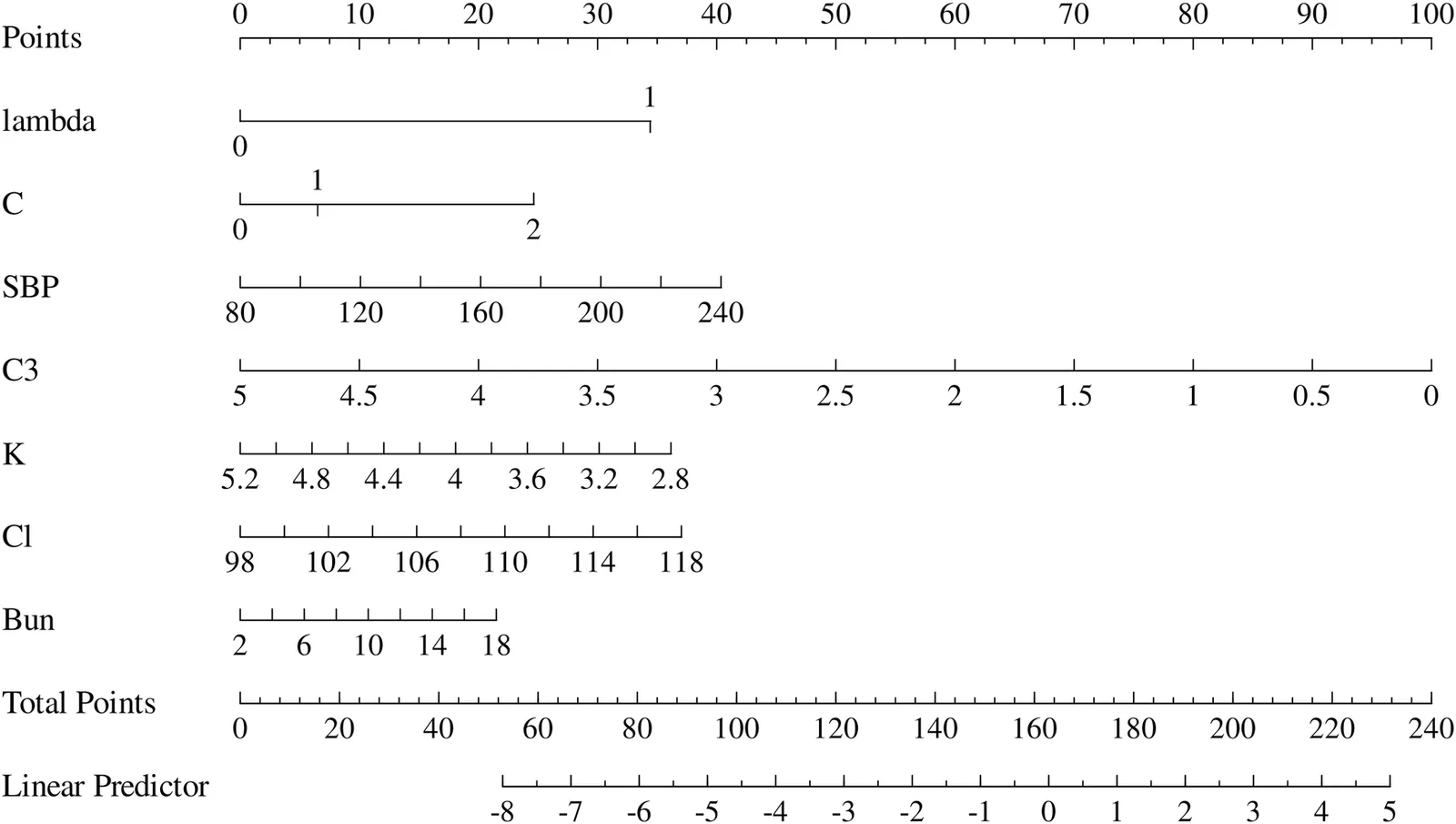
A nomogram model for assessing the risk of disease progression in patients with IgAN.

**Figure 3:**
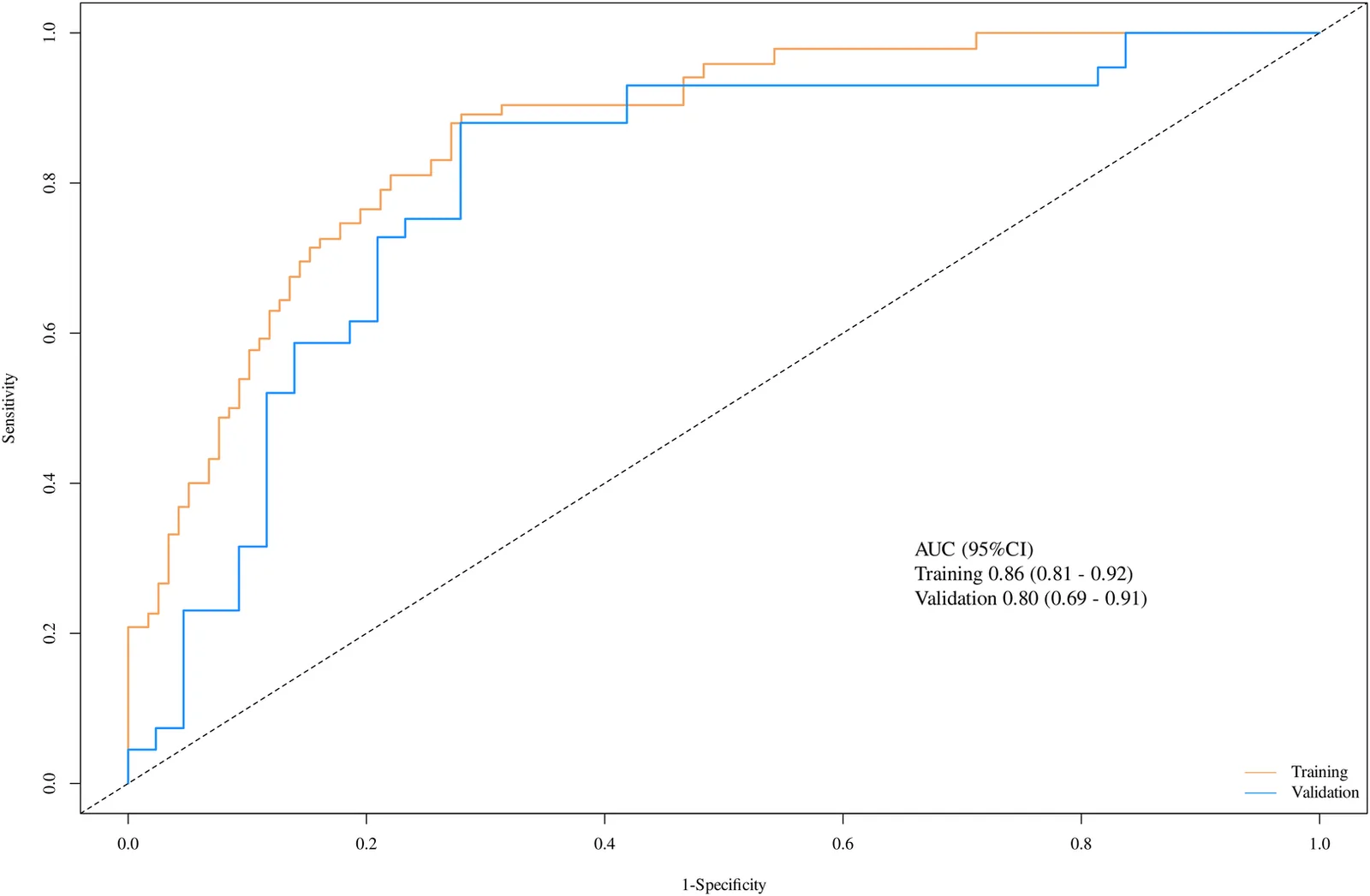
Receiver operating characteristic (ROC) curves for the training and validation cohorts, showing the 1-year time-dependent AUC at the 12-month time point of the time-to-event model.

**Figure 4:**
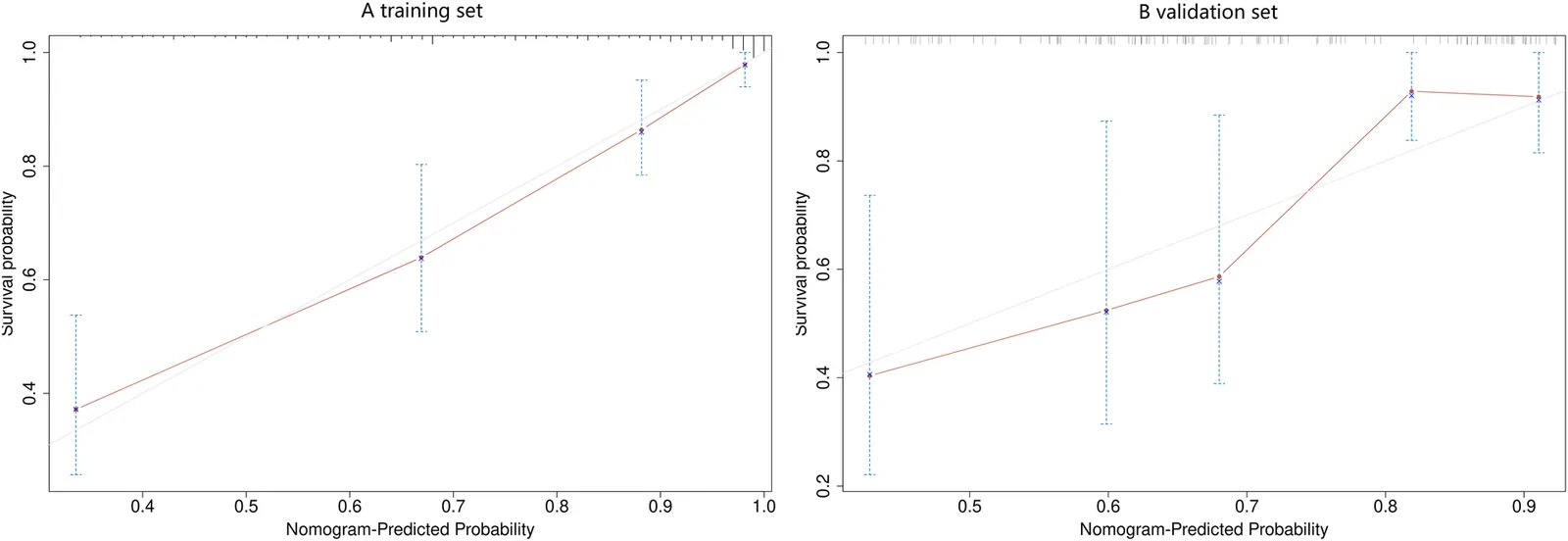
Calibration curves comparing predicted and observed 1-year risk of rapid renal function decline in the training **(A)** and validation **(B)** cohorts.

**Figure 5:**
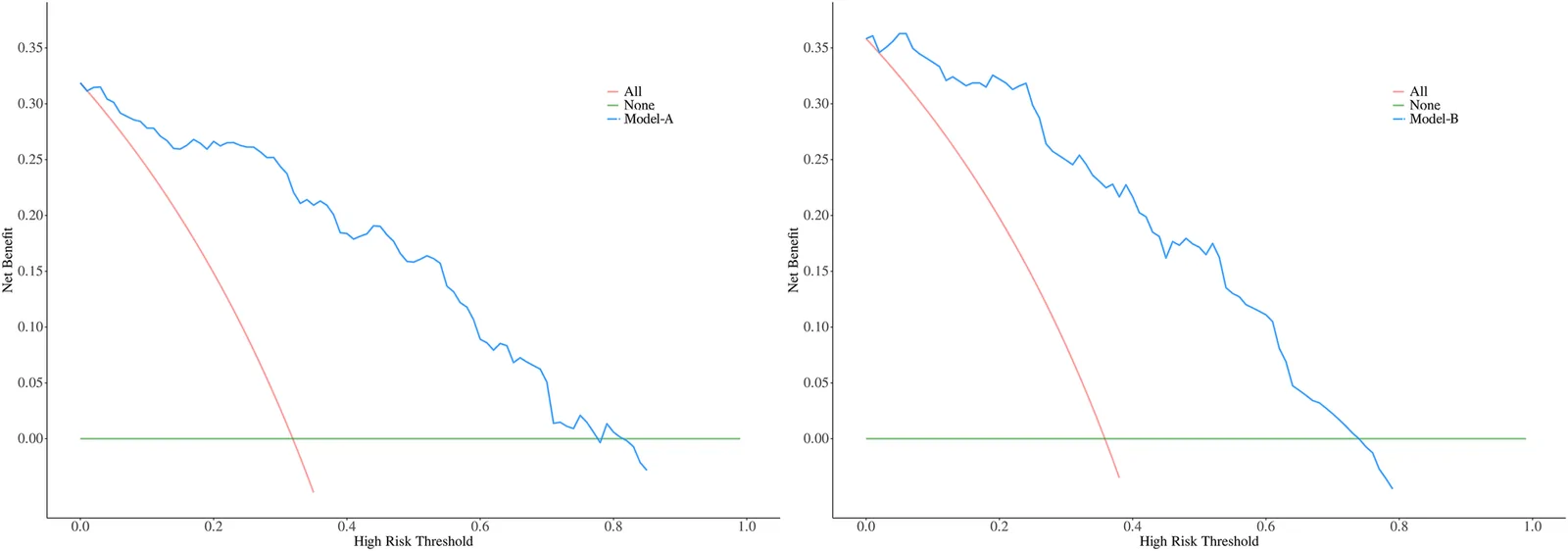
Decision curve analysis of net benefit for predicting 1-year risk of rapid renal function decline in the training **(A)** and validation **(B)** cohorts.

### 3.4 Model interpretability and feature importance analysis

To elucidate the contribution of each feature to the model's predictions, we used the SHAP (SHapley Additive exPlanations) framework. The mean absolute SHAP values were calculated to rank the global importance of the features. As shown in Figure 6A, Lambda light chain deposition was identified as the most influential feature driving the model's predictions, followed by serum complement C3, serum potassium, serum chloride, systolic blood pressure, Oxford C classification, and blood urea nitrogen. This ranking was broadly consistent with the hazard ratios observed in the multivariable analysis, reinforcing the central pathogenic role of Lambda light chain deposition in rapid renal function decline. The SHAP summary plot (Figure 6B) provided a granular view of how each feature value influenced the predicted risk for individual patients. In this visualization, each dot represents one patient. For Lambda light chain deposition, positive instances were predominantly distributed on the positive side of the SHAP axis, indicating that the presence of Lambda deposition consistently increased the predicted risk. A similar pattern was observed for serum chloride, systolic blood pressure, and blood urea nitrogen, for which higher values tended to be associated with higher predicted risk. In contrast, for serum complement C3 and serum potassium, higher values were mainly distributed on the negative side of the SHAP axis, supporting their protective effects in the model. These findings improve the interpretability of the model and provide clinicians with a transparent understanding of the relative contribution of each predictor.

**Figure 6:**
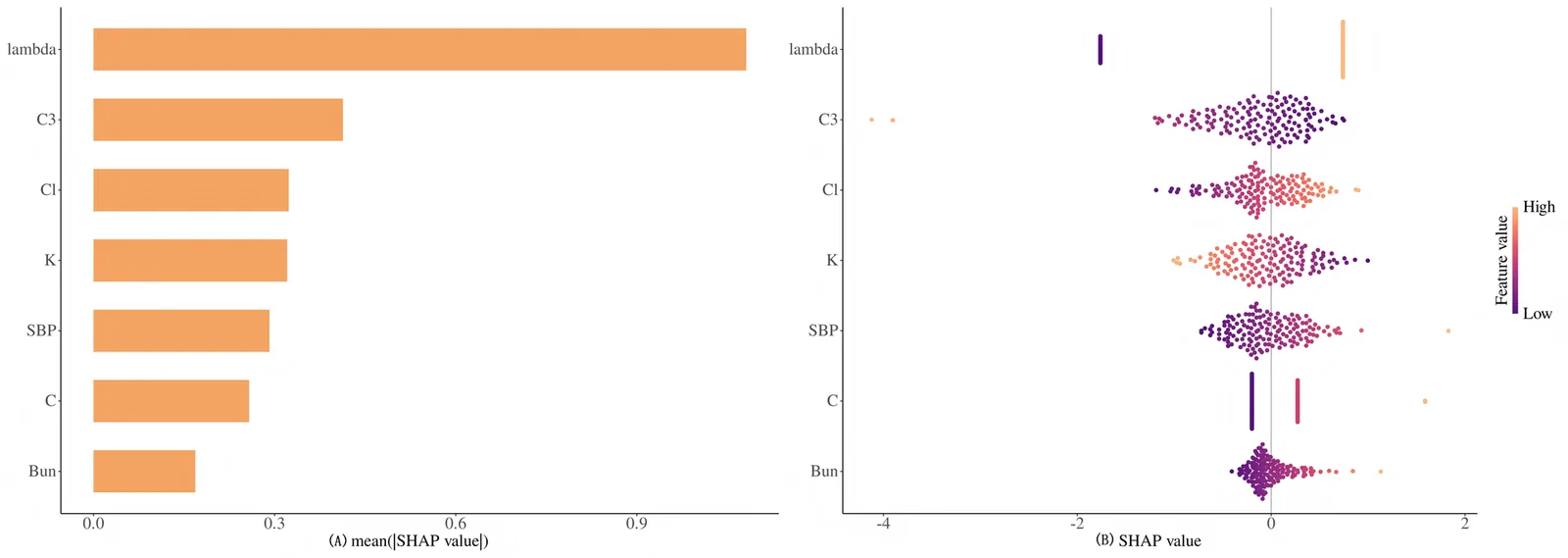
**(A)** presents the ranking of variable importance based on the mean absolute SHAP value (|SHAP value|), where higher values indicate a greater overall influence of the corresponding feature on the model's prediction outcomes; **(B)** presents the swarm plot illustrating the SHAP value distribution of the predicted features. The horizontal axis represents the SHAP values, indicating the direction and magnitude of each feature's influence on the predicted risk. The vertical axis lists the individual features. Each data point corresponds to a sample, with the color intensity reflecting the actual value of the respective feature for that sample (high values in dark color, low values in light color).

Kaplan–Meier analysis further confirmed clear separation of rapid-decline-free survival between the high- and low-risk groups stratified by the model's predicted risk in both cohorts (log-rank *p* < 0.001 in each cohort; Figure 7).

**Figure 7:**
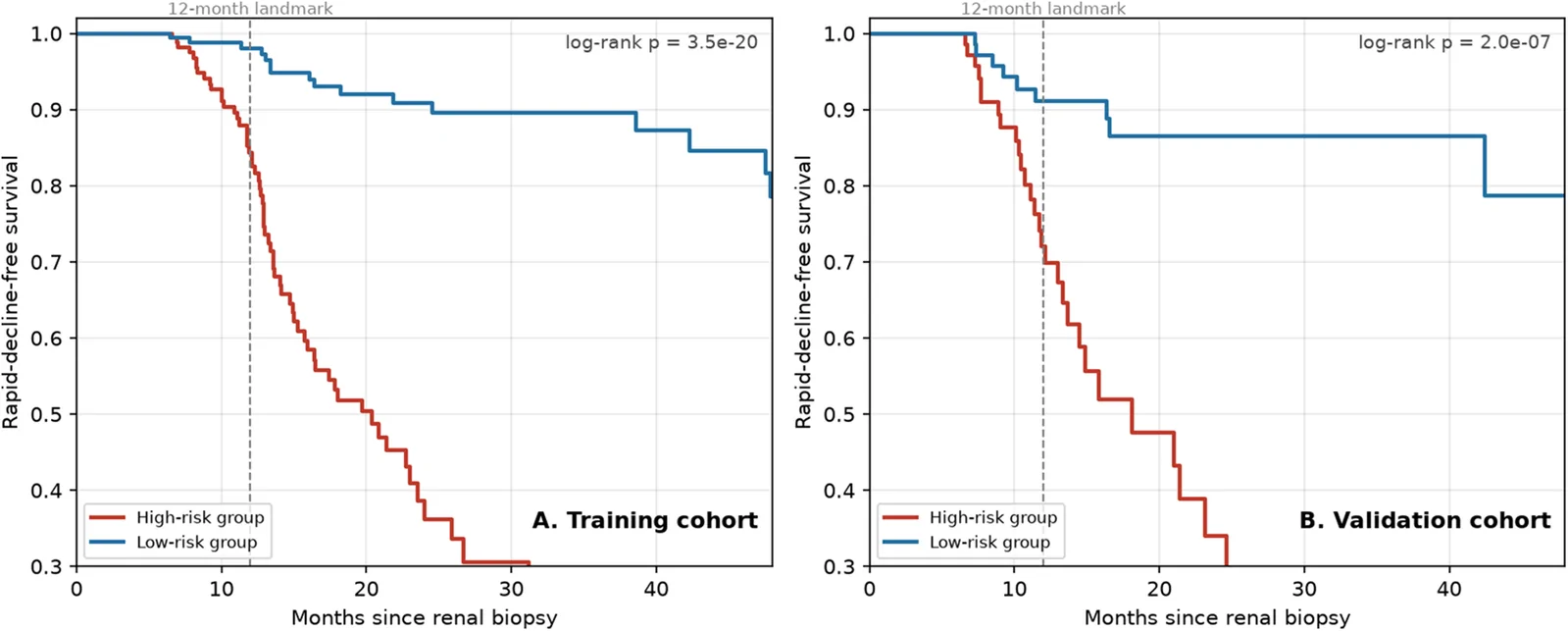
Kaplan–meier estimates of rapid renal function decline-free survival stratified by predicted risk group (high vs. low) in the training **(A)** and validation **(B)** cohorts. The 12-month landmark is indicated by the dashed vertical line; log-rank *p* < 0.001 in both cohorts.

These survival strata were defined by model-predicted risk (median split of the nomogram score), which is distinct from the observed-outcome-based grouping (progression vs. non-progression) used for the baseline characteristic comparisons in Table 2.

Notably, the high-/low-risk labels in Table 2 retain the observed-outcome definition (progression vs. non-progression) used for baseline comparison, whereas the stratification shown in Figure 7 follows the model-predicted risk for model validation; the two definitions differ in meaning but are applied in parallel, as they serve distinct purposes.

### 3.5 Sensitivity analyses

To examine whether the association between renal tissue Lambda light chain deposition and rapid renal function decline was confounded by baseline renal function or therapeutic regimen, we performed sensitivity analyses in the training cohort. The final multivariable model was sequentially re-fitted with additional adjustment for (i) eGFR, (ii) CKD stage (stage 2/3 vs. stage 1), and (iii) use of RAS inhibitors, glucocorticoids, and immunosuppressants. The hazard ratio for Lambda deposition remained essentially unchanged and highly significant across all scenarios (base model HR 12.21, 95% CI 4.56–32.69; +eGFR 12.50, 4.62–33.80; +CKD stage 12.62, 4.68–34.05; +medications 13.20, 4.81–36.17; +CKD stage and medications 13.83, 5.02–38.08; all *P* < 0.001). eGFR itself was not significantly associated with the outcome after adjustment (HR 0.998, 95% CI 0.988–1.009, *P* = 0.76). We further tested for effect modification by including product terms between Lambda deposition and CKD stage or medication use; neither interaction was statistically significant (CKD stage × Lambda: likelihood-ratio test *P* = 0.069; medication × Lambda: *P* = 0.549), indicating that the prognostic effect of Lambda deposition was consistent across renal-function strata and treatment groups. These analyses indicate that the observed Lambda–outcome association is not attributable to confounding by baseline renal function or therapeutic regimen (Supplementary Table S1). In exploratory subgroup analyses on the full cohort (*n* = 483), the association remained significant in the majority of pre-specified strata defined by CKD stage, RAS-inhibitor use, glucocorticoid use, immunosuppressant use, and eGFR (<60 vs. ≥60 mL/min/1.73 m^2^) (Supplementary Table S2).

## 4 Discussion

This multicenter retrospective study developed and validated a nomogram to predict the risk of rapid renal function decline, defined as *a* ≥ 25% reduction in eGFR or progression to ESRD, analyzed as a time-to-event outcome (with 1-year risk as the primary reported metric), in patients with early-stage IgA nephropathy (CKD stages 1–3) (9). Our analysis of 483 biopsy-proven cases identified seven independent predictors, including renal tissue Lambda light chain deposition, Oxford C score, systolic blood pressure, serum chloride, and blood urea nitrogen as risk factors, whereas serum complement C3 and potassium levels were identified as protective factors. The resulting model demonstrated good discrimination and calibration in both training and validation cohorts. A pivotal finding of this study is the identification of Lambda light chain deposition as the most potent predictor of rapid progression, with a hazard ratio of 12.21. This observation suggests that in patients with rapidly progressive IgAN, lambda light chain deposition may be associated with increased risk of glomerular damage.

Nevertheless, the exceptionally high hazard ratio (HR 12.21) with its correspondingly wide confidence interval should be interpreted cautiously: only a small number of lambda-negative patients experienced the endpoint, limiting estimation precision, and this finding requires confirmation in larger external cohorts. In comparison with established IgAN risk tools, our nomogram is, to our knowledge, among the first to incorporate mesangial Lambda light-chain deposition as a predictor of short-term (1-year) rapid decline. Widely used instruments such as the International IgAN Prediction Tool (Barbour et al., JAMA Intern Med 2019) (10) and MEST-C–based risk scores target longer-term endpoints (ESRD or 50% eGFR decline) and do not capture light-chain phenotype. The present model therefore complements rather than replaces these tools by adding a routinely available pathological biomarker oriented to early progression, where timely therapeutic intensification is most actionable.

The strong association between Lambda light chain deposition and poor prognosis observed in our cohort aligns with emerging evidence regarding the structural and functional role of light chains in IgAN (11). While IgAN is fundamentally defined by the deposition of galactose-deficient IgA1 (Gd-IgA1) (11), for which specific immunohistochemical detection (e.g., KM55) has refined diagnostic and prognostic assessment (42), recent investigations indicate that the light chain component critically influences the physicochemical properties of these immune complexes (12, 13); consistently, circulating Gd-IgA1-producing B cells preferentially carry lambda light chains and mucosal homing receptors (43). Biochemically, Lambda-IgA1 complexes exhibit a distinct anionic surface charge compared to Kappa-IgA1. Although the glomerular basement membrane typically repels negatively charged molecules (14), the specific electronegative character of Lambda-IgA1 may facilitate non-classical electrostatic interactions or expose hydrophobic regions of the heavy chain (15). These conformational changes likely enhance the affinity of the immune complex for mesangial receptors, such as the transferrin receptor CD71 (16, 17), thereby promoting selective mesangial retention and subsequent inflammatory signaling (18).

The prognostic significance of Lambda light chain dominance remains a subject of debate in the international literature, likely due to regional heterogeneity in genetic backgrounds and differences in pathological definitions (2). Our findings corroborate data from other large Asian cohorts, such as the study by The First Affiliated Hospital of Wenzhou Medical University, which identified mesangial Lambda restriction as an independent risk factor for end-stage renal disease (19). In contrast, Western cohorts, such as the CureGN study (11, 20), have reported no significant association between Lambda dominance and long-term kidney survival. This discrepancy may be attributed to the higher frequency of IgAN-susceptibility risk alleles in Asian populations, which might amplify the pathogenicity of Gd-IgA1 (21). Our study suggests that in the Asian population, Lambda deposition is a marker of a biologically aggressive subset of IgAN that warrants intensive monitoring.

Our results also revealed an association between Lambda light chain deposition and lower serum C3 levels. In our multivariate model, lower serum C3 levels were independently associated with rapid progression. Unlike hypocomplementemia commonly observed in diseases such as lupus nephritis (22), serum C3 levels in patients with IgA nephropathy rarely fall below the lower limit of normal. However, multiple studies have demonstrated that relatively low C3 levels within the normal range reflect localized inflammatory activity and are often associated with a more aggressive disease course (23, 24). This finding is consistent with the relative hypocomplementemia frequently seen in aggressive disease phenotypes (25). Previous mechanistic studies support a direct synergistic relationship between Lambda chains and complement activation (26). Lambda-IgA1 complexes demonstrate significantly higher binding affinity for Mannose-Binding Lectin (MBL) compared to Kappa-containing complexes (27). This enhanced binding triggers the lectin pathway, leading to the activation of mannan-binding lectin-associated serine protease-2 (MASP-2) and the subsequent cleavage of C4 and C2. The downstream effect is the formation of the C3 convertase and the robust activation of the alternative pathway amplification loop (28), resulting in local inflammation and glomerular injury. Consequently, the simultaneous observation of Lambda deposition and lower serum C3 in our high-risk cohort likely reflects a pathogenic axis where Lambda-containing complexes act as a potent scaffold for complement consumption and tissue damage.

The clinical implications of identifying Lambda light chain deposition as a major risk factor are substantial. First, it emphasizes the need for precise pathological subtyping. Although our exclusion criteria removed secondary causes, clinicians should remain vigilant and utilize serum free light chain ratios and IgG subclass staining to rule out underlying clonal disorders in Lambda-restricted cases. Second, the “Lambda-MBL-Complement” axis highlights potential therapeutic targets. The identification of Lambda light chain deposition as a critical pathogenic driver supports a paradigm shift toward mechanism-based precision medicine. Since Lambda-restricted Gd-IgA1-secreting plasma cells originate from gut-associated lymphoid tissue (29), targeted-release budesonide offers a strategic approach by suppressing pathogenic IgA production directly at its mucosal source (30). Similarly, upstream interventions targeting B-cell survival factors, including B-cell activating factor (BAFF) and a proliferation-inducing ligand (APRIL) inhibitors, hold promise for depleting these specific mucosal-homing lineages (31). For downstream blockade, proximal complement inhibitors targeting MASP-2 or Factor B are theoretically optimal for interrupting the lectin pathway activation specifically driven by Lambda-IgA1 complexes (32), potentially overcoming resistance to broad-spectrum immunosuppression. Thus, integrating light chain phenotyping into clinical practice could serve as a vital biomarker to guide the selection of these novel biological agents. However, as this is an observational study, the identified associations should not be interpreted as causal relationships.

Consistent with the Oxford classification system, our study confirmed that crescent formation (C score) is a robust predictor of short-term deterioration. This histological feature represents the structural consequence of severe glomerular inflammation and capillary wall rupture (33). Notably, prior research has demonstrated a statistical correlation between Lambda light chain dominance and active histological lesions, including endocapillary hypercellularity and crescents (34). This association further supports the hypothesis that Lambda-containing immune complexes possess a higher pro-inflammatory potential, capable of recruiting leukocytes and inducing necrosis (34). Therefore, the inclusion of both the Oxford C score and Lambda deposition in our nomogram captures both the upstream molecular trigger and the downstream histological damage, enhancing the predictive precision of the model.

Beyond immunological markers, our analysis identified metabolic and hemodynamic parameters, specifically serum potassium and chloride, as novel predictors of short-term progression. While systolic blood pressure and blood urea nitrogen are established risk factors reflecting glomerular hemodynamics and filtration capacity (35), and robust evidence supports intensive blood pressure control as a critical determinant of improved prognosis and treatment stabilization (36), the role of electrolytes in early IgAN is less characterized (37). In our model, higher serum potassium levels were protective, whereas elevated serum chloride levels were associated with increased risk. Lower serum potassium and higher chloride may serve as surrogate markers of tubulointerstitial dysfunction or dysregulation of the renin-angiotensin-aldosterone system (RAAS). Aldosterone-mediated sodium retention and potassium excretion are hallmark features of RAAS activation, a key driver of renal fibrosis (38). Thus, electrolyte imbalances in early-stage disease might signal an activated intrarenal RAAS or early tubular acidosis, preceding an overt decline in eGFR.

There are several limitations to this study. First, the retrospective design carries inherent risks of selection bias. Although treatment was assigned per guideline-directed care rather than randomized, we formally assessed potential confounding by baseline renal function and therapeutic regimen. Sensitivity analyses with additional adjustment for eGFR, CKD stage, and medication use, together with effect-modification tests, showed that the association between Lambda light chain deposition and rapid progression was robust and not explained by these factors (Supplementary Table S1). We nonetheless acknowledge that only baseline treatment (not time-updated regimen, intensity, or duration) was available, so residual confounding from unmeasured or time-varying treatment effects cannot be fully excluded. Second, our study population was exclusively Chinese, which limits the generalizability of the findings to non-Asian populations, particularly given the known ethnic variability in IgAN phenotypes (39). Third, while we established the prognostic value of Lambda deposition, we did not routinely measure serum or urinary free light chains, preventing us from evaluating their utility as non-invasive biomarkers. Future prospective studies should address the aforementioned deficiencies and investigate whether the level of circulating free light chains is associated with renal deposition and disease activity. Fourth, because the training and validation sets originated from a single pooled cohort by random split, the performance estimates reflect internal validation only; external validation in independent multicenter cohorts is required before generalizability can be claimed. In addition, patients with follow-up shorter than 12 months who were censored may have led to underestimation of the true event rate.

In conclusion, we have developed an applicable nomogram that provides a useful tool for estimating the risk of rapid renal function decline in early-stage IgAN. Our findings underscore the critical pathogenic role of Lambda light chain deposition, likely through mechanisms involving enhanced charge-dependent mesangial trapping and robust activation of the lectin complement pathway (40). By integrating pathological features with routine clinical markers, this tool facilitates the early identification of high-risk patients who may benefit from intensified immunosuppressive therapy or emerging complement-targeted interventions. This approach supports a shift towards precision medicine in the management of IgA nephropathy.

## 5 Conclusion

We developed and internally validated a nomogram for predicting rapid renal function decline in early-stage IgA nephropathy. The model showed good discrimination and calibration, and the inclusion of Lambda light chain deposition improved risk stratification. This tool may help support early individualized clinical management.

## Data Availability

The original contributions presented in the study are included in the article/Supplementary Material, further inquiries can be directed to the corresponding author/s.

## Supplementary material

Supplementary Table S1 Sensitivity analyses assessing confounding of the renal tissue Lambda light chain deposition effect by baseline renal function and therapeutic regimen (training cohort, n = 338).

Supplementary Table S2 Exploratory subgroup analyses of the renal tissue Lambda light chain deposition effect on rapid renal function decline in the full cohort (n = 483, events = 126).
