# Supplementary material for: Development and validation of a predictive model for rapid renal function decline in early IgA nephropathy: a multicenter retrospective study
Source: Front Med (Lausanne). 2026 Sep 24;13:1857665. doi: 10.3389/fmed.2026.1857665 (PMC13645597; doi:10.3389/fmed.2026.1857665)
Supplement: Supplementary file 1 [file Table1.docx]

**Supplementary Material**

*Development and Validation of a Predictive Model for Rapid Renal Function Decline in Early IgA Nephropathy:A Multicenter Retrospective Study*

**Supplementary Table S1. Sensitivity analyses assessing confounding of the renal tissue Lambda light chain deposition effect by baseline renal function and therapeutic regimen (training cohort, n = 338, events = 83).**

The final multivariable Cox model (7 predictors: SBP, C3, K, Cl, BUN, Lambda deposition, Oxford C score) was re-fitted with sequential additional adjustment. Hazard ratios (HR) and 95% confidence intervals (CI) for Lambda deposition are shown; Efron handling of ties was used throughout. P values are two-sided.

| **Model** | **Lambda HR (95% CI)** | **P value** | **Additional covariate HR (95% CI), P value** |
| --- | --- | --- | --- |
| Final model (7 predictors) | 12.21 (4.56–32.69) | <0.001 | — |
| + eGFR | 12.50 (4.62–33.80) | <0.001 | eGFR 0.998 (0.988–1.009), P = 0.76 |
| + CKD stage | 12.62 (4.68–34.05) | <0.001 | CKD-2 vs 1: 1.22 (0.67–2.24), P = 0.51; CKD-3 vs 1: 1.10 (0.51–2.37), P = 0.81 |
| + Medications | 13.20 (4.81–36.17) | <0.001 | RAS 1.67 (0.97–2.89), P = 0.064; GC 1.12 (0.68–1.83), P = 0.66; IS 1.80 (1.11–2.90), P = 0.016 |
| + CKD stage + medications | 13.83 (5.02–38.08) | <0.001 | CKD-2/3 and RAS/GC/IS as above (eGFR omitted: collinear with CKD stage) |

Effect-modification (interaction) tests in the training cohort — likelihood-ratio tests comparing models with vs without product terms between Lambda deposition and the listed factor:

| **Interaction term** | **LR χ²** | **df** | **P value** |
| --- | --- | --- | --- |
| CKD stage × Lambda deposition | 5.36 | 2 | 0.069 |
| Medication use × Lambda deposition | 2.12 | 3 | 0.549 |

*Neither interaction was statistically significant, indicating that the prognostic effect of Lambda deposition was consistent across renal-function strata and treatment groups. CKD stage was coded as stage 2 and stage 3 versus stage 1 (reference); medication use indicates RAS inhibitors, glucocorticoids, and immunosuppressants (each binary, yes/no). GC = glucocorticoids; IS = immunosuppressants; RAS = renin–angiotensin system.*

**Supplementary Table S2. Exploratory subgroup analyses of the renal tissue Lambda light chain deposition effect on rapid renal function decline in the full cohort (n = 483, events = 126).**

Within each subgroup, a Cox model was fitted for Lambda deposition adjusted for the other six predictors of the final model. Hazard ratios (HR) and 95% CIs are reported.

| **Subgroup** | **n** | **Events** | **Lambda HR (95% CI)** | **P value** |
| --- | --- | --- | --- | --- |
| CKD stage 1 | 165 | 31 | 4.55 (1.01–20.58) | 0.049 |
| CKD stage 2 | 189 | 50 | 19.46 (5.31–71.30) | <0.001 |
| CKD stage 3 | 129 | 45 | 4.28 (1.66–11.05) | 0.003 |
| RAS inhibitor: yes | 311 | 92 | 6.93 (2.93–16.36) | <0.001 |
| RAS inhibitor: no | 172 | 34 | 10.09 (2.93–34.72) | <0.001 |
| Glucocorticoids: yes | 175 | 61 | 6.05 (2.26–16.17) | <0.001 |
| Glucocorticoids: no | — | — | omitted (model did not converge) | — |
| Immunosuppressant: yes | 148 | 48 | 5.36 (2.02–14.22) | <0.001 |
| Immunosuppressant: no | 335 | 78 | 13.26 (4.86–36.20) | <0.001 |
| eGFR ≥ 60 mL/min/1.73 m² | 353 | 81 | 11.18 (4.09–30.55) | <0.001 |
| eGFR < 60 mL/min/1.73 m² | 130 | 45 | 4.46 (1.72–11.56) | 0.002 |

*These analyses are exploratory. The glucocorticoid non-user stratum was omitted because the Cox model did not converge (sparse events within a covariate pattern). The point estimate for CKD stage 2 (HR 19.46) has a wide confidence interval and should be interpreted with caution as hypothesis-generating.*
